# Sternal Wound Reconstruction With Omental Flap for Poststernotomy Mediastinitis

**Published:** 2013-02-18

**Authors:** Laurel Karian, Mark Granick

**Affiliations:** Division of Plastic Surgery, University of Medicine and Dentistry of New Jersey, Newark.

## DESCRIPTION

**Figure 1:**
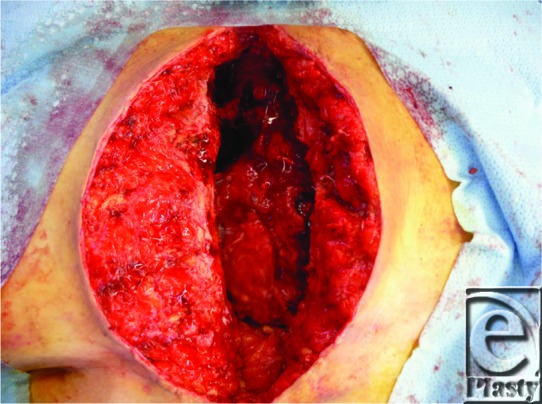
Mediastinal defect with exposed prosthetic aortic root graft.

A 70-year-old woman with short stature and hypermastia underwent ascending aortic aneurysm repair with a prosthetic graft, complicated by postoperative mediastinitis and sternal dehiscence. She was treated with aggressive sternal debridement and negative pressure wound therapy for 1 week (Fig 1) prior to definitive closure with an omental transposition flap and eventual skin graft to the sternal wound. Her hospital course prior to sternal wound closure was also complicated by a deep venous thrombosis (DVT) and pulmonary embolus (PE) for which she was treated with anticoagulation.

## QUESTIONS

1. **What are the requirements for debriding sternal wounds complicated by mediastinitis?**
2. **What is the role of negative pressure wound therapy in postoperative mediastinitis?**
3. **What are the options for reconstruction of sternal wounds in cardiothoracic surgery patients?**
4. **Why was the omental flap the best option for this particular patient?**

## DISCUSSION

Mediastinitis, or deep sternal wound infection, is a feared life-threatening complication of cardiac surgery involving a midline sternotomy, with an in-hospital mortality rate as high as 31%.^1^^,^^2^ Treatment of sternal wounds complicated by mediastinitis involves debriding the affected sternum with partial or total sternectomy followed by immediate or delayed closure. Sternal debridement requires removal of all nonviable and infected soft tissue, all the affected sternum and the sternal wires, and other foreign material. It is essential to perform careful debridements to avoid injuring vital structures. In subacute wounds, the mediastinum is often healed to the underside of the sternum, making the right ventricle prone to tears during sternal debridement. Soft tissue and bone cultures should be obtained intraoperatively to determine appropriate antibiotics and extent of therapy. In complete sternectomy, the avascular costal cartilages are a potential source for infection and should be removed.

Negative pressure wound therapy is a highly effective treatment strategy for infected sternal wounds as a temporary dressing between debridements until the wound is clean enough for definitive closure.^3^ Negative pressure wound therapy acts to increase local blood flow, remove fluid and necrotic tissue, reduce bacterial count, accelerate wound contraction, and provide a moist environment for wound healing.^4^ Negative pressure wound therapy has been shown to reduce the time to definitive wound closure and the cost associated with prolonged hospital stay.^3^

**Figure 2:**
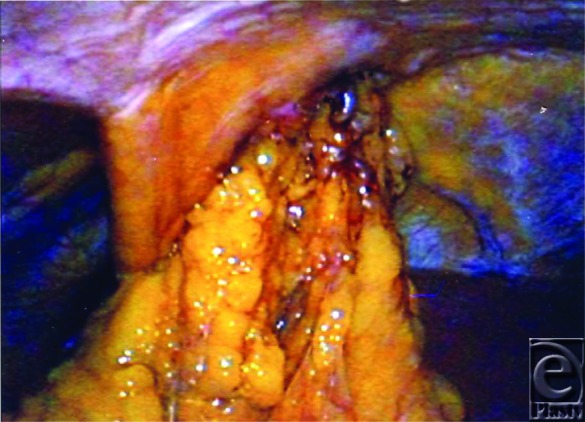
Laproscopic view of the omental flap being transposed through the diaphragm.

Options for reconstruction of sternal wounds include a unilateral pectoralis major muscle “turnover” flap, unilateral or bilateral pectoralis major muscle or myocutaneous advancement flap, rectus abdominis muscle or myocutaneous flap, latissimus dorsi muscle or myocutaneous pedicled or microvascular flap, omental flap, or a combination of these.^5^^,^^6^ Use of one or more pectoralis flaps is generally the first line for reconstruction of sternal wounds.^1^ A rectus abdominis transposition flap may be ideal for defects in the lower third of the mediastinum but requires an intact ipsilateral internal mammary artery. Rectus flaps cannot be used if bilateral internal mammary arteries have been harvested for grafts.^1^ The omental transposition flap has been shown to be effective in sternal wound reconstruction, especially in irregular defects or when muscle flaps have failed.^5^ Omental flaps transposed through the diaphragm (Fig 2) are advantageous because of their robust blood supply, relatively long vascular pedicle enabling transfer to the anterior mediastinum, bulk, ability to cover irregular defects, and provision of lymphocytes and angiogenesic factors.^1^ They also readily accept a split-thickness skin graft without requiring an additional delay to wait for granulation tissue. A problem with omental flaps is the potential for patchy fat necrosis.

This particular case involved an exposed prosthetic aortic root graft and suture line lying deep within the mediastinum. A pectoralis or other muscle flap would have not been adequate to reach and envelop the graft suture line in the posterior mediastinum. Omentum is more malleable than muscle and has a longer pedicle. It also has a minimal raw surface and donor site, which is helpful in reducing hematoma risk in anticoagulated patients. Consequently, the omentum was the best choice to mold into and fill this deep mediastinal defect.

## Figures and Tables

**Figure 3:**
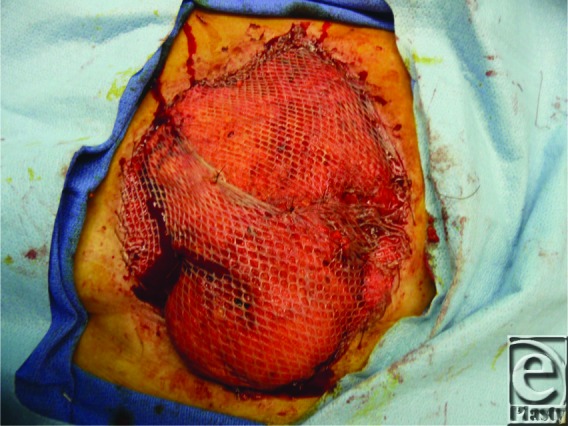
Mediastinal defect closed with omental flap and split-thickness skin graft.
